# HIV Screening Among Young Black Men Who Have Sex with Women in New Orleans, LA

**DOI:** 10.1007/s10461-024-04354-7

**Published:** 2024-05-07

**Authors:** Aneeka Ratnayake, Gérard Gomes, Patricia J. Kissinger

**Affiliations:** Department of Epidemiology, Tulane University School of Public Health and Tropical Medicine, 1440 Canal Street, Suite 2004, New Orleans, LA 70112 USA

**Keywords:** HIV, Screening, Deep south, Men who have sex with women

## Abstract

There are significant disparities in HIV acquisition, with Black individuals facing disproportionately more new diagnoses. Per Centers for Disease Control and Prevention (CDC), all people aged 13–64 should be tested at least once in their lifetime, and men at increased risk (e.g., those who have male sexual contact, multiple partners, have partners with multiple partners, or share drug injection equipment) should be tested annually. The study included young Black men who have sex with women (MSW), aged 15–26, and who live in New Orleans, LA. Survey data was used to elicit the frequency and factors associated with three self-reported outcomes: (1) history of ever HIV testing, (2) HIV screening in the last year among those who were recommended per CDC, and (3) HIV positivity. Of the 1321 men included, 694/1321 men (52.5%) reported ever having been HIV tested. There were 708/1321 (54.2%) men who met the recommendation for annual screening and 321/708 (45.3%) of these eligible men reported being tested in the previous year. Of those ever tested, 44/694 (6.3%) self-reported testing positive. In logistic regression analysis, older age (OR: 1.27, *p* < 0.001), prior STI testing (OR: 6.45, *p* < 0.001), and prior incarceration (OR:1.70, *p* = 0.006) were positively associated with having ever received an HIV test, and ever having a male partner (OR: 3.63, *p* = 0.014) was associated with HIV positivity. Initiatives to improve HIV testing rates among young Black men who have sex with women are needed to reduce the burden of HIV and help the End the Epidemic initiative.

**Supplementary Information:**

The online version contains supplementary material available at 10.1007/s10461-024-04354-7.

## Introduction

HIV continues to cause significant morbidity and mortality in the United States, particularly in the Deep South with undiagnosed infection in cisgender heterosexuals. Though the majority of HIV transmission in the US occurs through male-to-male sexual contact, heterosexual contact was still responsible for roughly 22% of new infections in 2021 [1]. HIV can be treated, and sustained treatment can lead to viral suppression, wherein people who are living with HIV can no longer transmit the virus [2]. Hence, prompt screening for HIV is a critical first step to ensuring individuals are aware of their status, and receiving treatment, thus minimizing sequelae and further transmission [3, 4]. 

The CDC recommends that all people between 13 and 64 be screened at least once in their lifetime, and that those at elevated risk be screened annually [5]. Factors associated with elevated HIV risk for men include having had sex with a man, having had anal or vaginal sex with someone who has HIV, having had more than one sex partner since their prior HIV test, sharing drug injection equipment, exchanging money for sex, having a prior STI diagnosis, and being diagnosed or treated for hepatitis or tuberculosis [6]. 

Young Black men face a disproportionate burden of HIV, compared to White men. In 2019, HIV rates were eight times higher among Black men than White men, and the largest share of new HIV diagnoses were among Black men aged 25–34 followed by Black men aged 13–24 [7]. Moreover, the rate of HIV is particularly elevated in the Deep South compared to other geographic regions, for a number of reasons including structural racism, greater stigma around sexuality, and lesser access to sex education and HIV services [8–10]. The Deep South accounts for 51% of new HIV cases annually, despite only having 38% of the US population [11]. In Louisiana, an estimated 15.7-18.2% of people who are living with HIV are unaware of their status compared to 14% in the US as a whole, suggesting that more people need to get screened [11, 12]. Hence, understanding predictors of HIV testing among young Black men at elevated risk residing in the Deep South is critical to mitigating the impact of HIV on this population.

The goals of the present study were to examine three self-reported outcomes: (1) history of ever HIV testing and factors associated with this screening, (2) prevalence of HIV screening in the last year among those who were eligible as well factors associated with this screening, and (3) prevalence of HIV positive status and factors associated with HIV positivity.

## Methods

Data from the *Check It* study were used. The *Check it* study was a community based seek, test, and treat *Chlamydia trachomatis* (Ct) and *Neisseria gonorrhoeae* (GC) screening program for Black or African American identifying men who have sex with women aged 15–26 years old in New Orleans, LA. The goal of this program was to determine whether men should be included in CDC screening guidelines for Ct. Methods have been described elsewhere [13] but briefly, the cross-sectional investigation was designed to evaluate the effectiveness of the program in reducing Ct infection rates in young Black women aged 15–26 by screening their male peer group in non-clinical, community venues and providing patient delivered expedited partner treatment options to Ct positive participants. The study was conducted between 3/2018 and 3/2020. The study found that testing young men reduced rates in women in a cost-effective manner [14, 15]. 

To participate, individuals had to report that they had a penis (i.e., were a cisgender man) at the time of intervention, were between the ages of 15 to 26 years, were African American or Black, had previously engaged in vaginal sex at some time in their life, lived in or frequented Orleans Parish (County), were able to speak and understand English, had not taken azithromycin in the two weeks before enrollment, and had not previously enrolled in *Check It*.

After eligibility was verified for interested participants, written consent was obtained. After this, participants underwent a computer-assisted, self-administered survey captured on Qualtrics that elicited demographic information, information on sexual behaviors, and partner-level information. Participants then provided a first-catch urine sample for Ct/GC testing. Participants who tested positive for Ct and/or GC were contacted with their results and offered treatment options for themselves and their partners. This study was approved by the Tulane University IRB and registered on Clinical Trials.gov. The study did not test for HIV but the survey elicited information about HIV testing and results.

In the present study, questions about HIV testing history were used, along with information related to demographics, sexual risk behaviors, and testing Ct or GC positive during the study. Odds ratios were computed to determine the association of selected factors with lifetime HIV test receipt, HIV testing in the previous year, and HIV positivity. Multivariable logistic regression models were then used to assess confounding. Factors were selected based on literature around known risk factors for HIV, and barriers and facilitators to testing.

The proportion of those tested in the past year according to guidance was also calculated. Those who had male sexual contact, multiple partners, or had partners with multiple partners, were classified as being recommended for annual testing, per CDC guidelines. Additionally, reporting injecting drugs was used as a proxy for possibly sharing injection equipment given that this question was not asked in the survey; individuals reporting the use of injection drugs were classified as recommended for annual testing. Anyone without these risk factors was classified as not recommended for annual testing [6]. 

## Results

There were 1321 men with information on HIV testing who were included in the sample; 29 men (2.1%) were excluded for nonresponse. The median age was 19.7 (range: 15.0-25.6), and the vast majority (97.0%) were of Black race only, with 3% identifying as being of more than one race including Black race. Over a third (39.2%) reported more than a high school education, and the majority (79.7%) had some form of health insurance. Binge drinking was reported by only 26.9% of participants, though most (62.7%) reported non-medical drug use. Of those reporting drug use, the majority was marijuana use only (85.5% of those reporting any drug use). Nearly a fifth (19.2%) of participants had previously been incarcerated. The median number of lifetime sexual partners was 5 (range 1–90), and the majority of individuals (63.0%) only reported one female sex partner in the previous two months. Forty-eight men (3.6%) reported having had a male partner. Fewer than half of individuals (41.6%) reported previously receiving a Ct test, and testing was even lower for GC (25.4%) and syphilis (17.7%). One in ten (10.6%) men tested positive for Ct and/or GC during the study. Full demographic information is presented in Table 1.

**Table 1:** Demographic Factors of Sample (*n* = 1321)

Variable	*N*(%) unless otherwise specified
Age (median, range)	19.7 (15.0-25.6)
Race	
Black only	1282 (97.0%)
More than one race	39 (3.0%)
Ethnicity	
Hispanic	43 (3.3%)
Non-Hispanic	1277 (96.7%)
Education	
High school graduate or less	801 (60.8%)
More than high school	516 (39.2%)
Has a form of insurance	968 (79.7%)
Used government assistance in past year	475 (37.1%)
Binge drinking in past 2 months	355 (26.9%)
Drug use in past 2 months	828 (62.7%)
Marijuana Use Only in past 2 months	708 (54.5%)
Age at sexual debut (median, range)	15 (13–24)
Consistent Condom Use in past 2 months	505 (38.2%)
Number of Female Sexual Partners	
One female sexual partner in past 2 months	832 (63.0%)
Two or more female sexual partners in 2 months	489 (37.0%)
Total Lifetime Sexual Partners (median, range)	5 (1–90)
Male Partners in Lifetime	48 (3.6%)
Ever incarcerated	254 (19.2%)
Prior Ct test	550 (41.6%)
Prior GC test	240 (25.4%)
Prior syphilis test	167 (17.7%)
Ct/GC positive during study	148 (11.2%)
HIV Test in Lifetime	694 (52.5%)
HIV Test in Part Year	117 (10.6%)
HIV Positivity (self-reported)	44 (6.5%)

Of all men included, 694/1321 (52.5%) self-reported ever having been HIV tested. Of those ever tested, 44/694 (6.3%) self-reported testing positive. Of the 708/1321 (54.2%) men who met the recommendation for annual testing only 321/708 (45.3%) reported being tested in the previous year.

In bivariate analyses, lifetime HIV testing was associated with older age (OR: 1.34 per year older, *p* < 0.001), having more than a high school education (OR:1.85, *p* < 0.001), drug use (OR: 1.58, *p* < 0.001), marijuana use only (OR:1.51, *p* < 0.001), binge drinking (OR:1.70, *p* < 0.001), inconsistent condom use (OR:1.71, *p* < 0.001), multiple partners in the past two months (OR: 1.28, *p* = 0.029), having more lifetime female partners (OR: 1.04 per additional partner *p* < 0.001), former incarceration (OR:2.00, *p* < 0.001), prior Ct testing (OR: 8.54, *p* < 0.001), prior GC testing (18.10, *p* < 0.001), and prior syphilis testing (OR:27.60, *p* < 0.001). Complete bivariate analyses are presented in Table 2. However, in multivariate analysis, only age (OR: 1.27 per year older, *p* < 0.001), prior Ct, GC, and/or syphilis testing (OR: 6.45, *p* < 0.001), and prior incarceration (OR: 1.70, *p* = 0.006) were associated with lifetime HIV test receipt. The multivariable logistic regression model is presented in Table S1.

**Table 2:** Selected Factors by Lifetime HIV Testing Status (*n* = 1321)

Variable	HIV Untested (*n* = 627)	HIV Tested (*n* = 694)	Unadjusted Odds Ratio	*P*-value
**Age** (median, range)	18.8 (15.1–25.2)	20.9 (15.0-25.6)	1.37 (1.31, 1.45)	**< 0.001**
**Race**				
Black only	606 (96.7%)	676 (97.4%)	1.00	0.418
More than one race	21 (3.3%)	18 (2.6%)	0.77 (0.41, 1.46)	
**Ethnicity**				
Hispanic	608 (97.1%)	669 (96.4%)	1.00	0.458
Non-Hispanic	19 (2.9%)	25 (3.6%)	1.26 (0.68, 2.34)	
**Education**				
High school graduate or less	427 (68.4%)	374 (54.0%)	1.00	**< 0.001**
More than high school	197 (31.6%)	319 (46.0%)	1.85 (1.48, 2.32)	
**Has a form of insurance**				
No	100 (17.8)	136 (22.4%)	1.00	**0.047**
Yes	462 (82.2%)	506 (77.6%)	0.75 (0.56, 0.99)	
**Used government assistance in past year**				
No	370 (61.9%)	435 (63.8%)	1.00	0.480
Yes	228 (38.1%)	247 (36.2%)	0.92 (0.73, 1.16)	
**Binge drinking in past 2 months**				
No	492 (78.5%)	472 (68.2%)	1.00	**< 0.001**
Yes	135 (21.5%)	220 (31.8%)	1.70 (1.32, 2.18)	
**Drug use in past 2 months**				
No	257 (41.9%)	215 (31.3%)	1.00	**< 0.001**
Yes	357 (58.1%)	471 (68.7%)	1.58 (1.26, 1.98)	
**Marijuana Use Only in past 2 months**				
No drug use	257 (45.1%)	215 (35.2%)	1.00	**< 0.001**
Yes	313 (54.9%)	395 (64.8%)	1.51 (1.19, 1.91)	
**Age at sexual debut** (median, range)	15.0 (13.0–23.0)	15.0 (13.0–24.0)	1.00 (0.95, 1.06)	0.395
**Consistent Condom Use in past 2 months**				
No	215 (54.7%)	401 (62.2%)	1.00	**0.008**
Yes	261 (45.3%)	244 (37.8%)	0.73 (0.58, 0.92)	
**Number of Female Sexual Partners in past 2 months**				
One female sexual partner	414 (66.0%)	418 (60.2%)	1.00	**0.029**
Two or more partners	213 (34.0%)	276 (39.8%)	1.28 (1.02, 1.61)	
**Total Lifetime Sexual Partners** (median, range)	4.0 (1.0–90.0)	6.0 (1.0–90.0)	1.04 (1.02, 1.05)	**< 0.001**
**Male Partners in Lifetime**				
No	592 (96.7%)	645 (95.8%)	1.00	0.396
Yes	20 (3.3%)	28 (4.2%)	1.29 (0.72, 2.31)	
**Prior Incarceration**				
No	521 (85.7%)	499 (74.9%)	1.00	**< 0.001**
Yes	87 (14.3%)	167 (25.1%)	2.00 (1.50, 2.67)	
**Prior Ct test**				
No	518 (82.9%)	251 (36.2%)	1.00	**< 0.001**
Yes	107 (17.1%)	443 (63.8%)	8.54 (6.59, 11.07)	
**Prior GC test**				
No	385 (96.3%)	319 (58.6%)	1.00	**< 0.001**
Yes	15 (3.8%)	225 (41.4%)	18.10 (10.51, 31.17)	
**Prior syphilis test**				
2003;No	394 (98.5%)	383 (70.4%)	1.00	**< 0.001**
Yes	6 (1.5%)	161 (29.6%)	27.60 (12.07, 63.11)	
**Ct/GC positive during study**				
No	565 (90.1%)	608 (87.6%)	1.00	0.150
Yes	62 (9.9%)	86 (12.4%)	1.29 (0.91, 1.82)	

Similarly, among those recommended for annual screening, in bivariate analyses, age (OR: 1.29 per year older, *p* < 0.001), having more than high school education (OR: 1.63, *p* = 0.002), binge drinking (OR: 1.51, *p* = 0.01), older age at sexual debut (OR: 1.08 per year older, *p* = 0.041), more lifetime sexual partners (OR: 1.02, *p* < 0.001), prior incarceration (OR: 2.00, *p* < 0.001), and having received a prior Ct (OR: 8.54, *p* < 0.001), GC (OR: 18.10, *p* < 0.001), or syphilis test (OR: 27.60, *p* < 0.001) were associated with receiving an HIV test in the past year. Complete bivariate analyses are presented in Table 3. However, in multivariable logistic regression, only age (OR: 1.19, *p* < 0.001) and receiving a Ct, GC, or syphilis test (OR: 6.00, *p* < 0.001) were statistically significant. The multivariable logistic regression model is presented in Table S2.

**Table 3:** Selected Factors by HIV Testing in Prior Year Among Those Recommended (*n* = 708)

Variable	HIV Untested in Past Year (*n* = 387)	HIV Tested in Past Year (*n* = 321)	Unadjusted Odds Ratio	*P*-value
**Age** (median, range)	19.1 (15.1–25.2)	20.9 (15.3–25.6)	1.29 (1.21, 1.38)	**< 0.001**
**Race**				
Black only	370 (956%)	314 (97.8%)	1.00	0.105
More than one race	17 (4.4%)	7 (2.2%)	0.48 (0.20, 1.18)	
**Ethnicity**				
Hispanic	376 (97.2%)	306 (95.3%)	1.00	0.197
Non-Hispanic	11 (2.8%)	15 (4.7%)	1.68 (0.76, 3.70)	
**Education**				
High school graduate or less	247 (64.0%)	167 (52.2%)	1.00	**0.002**
More than high school	139 (36.0%)	153 (47.8%)	1.63 (1.20, 2.20)	
**Has a form of insurance**				
No	62 (17.9%)	68 (22.1%)	1.00	0.171
Yes	285 (82.1%)	239 (77.9%)	0.76 (0.52, 1.12)	
**Used government assistance in past year**				
No	221 (59.2%)	194 (62.0%)	1.00	0.466
Yes	152 (40.8%)	119 (38.0%)	0.89 (0.66, 1.21)	
**Binge drinking in past 2 months**				
No	274 (70.8%)	197 (61.6%)	1.00	**0.010**
Yes	113 (29.2%)	123 (38.4%)	1.51 (1.11, 2.07)	
**Drug use in past 2 months**				
No	126 (33.0%)	85 (26.7%)	1.00	0.073
Yes	256 (67.0%)	233 (73.3%)	1.35 (0.97, 1.87)	
**Marijuana Use Only in past 2 months**				
No drug use	126 (36.1%)	85 (31.6%)	1.00	0.242
Yes	223 (63.9%)	184 (68.4%)	1.22 (0.87, 1.71)	
**Age at sexual debut** (median, range)	15.0 (13.0–24.0)	15.0 (13.0–24.0)	1.08 (1.00, 1.16)	**0.041**
**Consistent Condom Use in past 2 months**				
No	219 (61.7%)	189 (62.4%)	1.00	0.857
Yes	136 (38.3%)	114 (37.6%)	0.97 (0.71, 1.33)	
**Number of Female Sexual Partners in past 2 months**				
One female sexual partner	125 (32.3%)	94 (29.3%)	1.00	0.387
Two or more partners	262 (67.7%)	227 (70.7%)	1.15 (0.84, 1.59)	
**Total Lifetime Sexual Partners** (median, range)	5.0 (1.0–90.0)	8.0 (1.0–90.0)	1.02 (1.01, 1.04)	**< 0.001**
**Male Partners in Lifetime**				
No	352 (93.6%)	288 (92.3%)	1.00	0.502
Yes	24 (6.4%)	24 (7.7%)	1.22 (0.68, 2.20)	
**Prior Incarceration**				
No	306 (82.3%)	225 (73.1%)	1.00	**0.004**
Yes	66 (17.7%)	83 (26.9%)	1.71 (1.19, 2.47)	
**Prior Ct test**				
No	288 (74.4%)	112 (34.9%)	1.00	**< 0.001**
Yes	99 (25.6%)	209 (65.1%)	5.43 (3.93, 7.50)	
**Prior GC test**				
No	118 (88.7%)	28 (35.9%)	1.00	**< 0.001**
Yes	15 (11.3%)	50 (64.1%)	14.05 (6.91, 28.54)	
**Prior syphilis test**				
No	236 (93.7%)	157 (65.1%)	1.00	**< 0.001**
Yes	16 (6.3%)	84 (34.9%)	7.89 (4.46, 13.97)	
**Ct/GC positive during study**				
No	340 (87.9%)	276 (86.0%)	1.00	0.460
Yes	47 (12.1%)	45 (14.0%)	1.18 (0.76, 1.83)	

Being of younger age (OR: 0.87 per year older, *p* = 0.042), having a male partner (OR:3.63, *p* = 0.009), and prior incarceration (OR: 1.92, *p* = 0.045) were positively associated with HIV positivity in bivariate analyses and binge drinking (OR: 0.46, *p* = 0.047) was inversely associated with HIV positivity in bivariate analyses. However, in multivariable logistic regression, only having a male partner was significantly associated with HIV positivity (OR: 3.63, *p* = 0.014). Full statistics are presented in Table 4 and S3.

**Table 4:** Selected Factors by HIV Positivity Status (*n* = 694)

Variable	HIV Negative (*n* = 650)	HIV Positive (*n* = 44)	Unadjusted Odds Ratio	*P*-value
**Age** (median, range)	20.9 (10.5)	19.6 (9.8)	0.87 (0.76, 0.99)	**0.042**
**Race**				
Black only	631 (97.2%)	44 (100%)	0.93 (0.92, 0.95)	0.621
More than one race	18 (2.8%)	0 (0%)		
**Ethnicity**				
Hispanic	628 (96.8%)	41 (93.2%)	2.19 (0.63, 7.64)	0.209
Non-Hispanic	21 (3.2%)	3 (6.8%)		
**Education**				
High school graduate or less	350 (53.9%)	24 (55.8%)	0.93 (0.50, 1.72)	0.810
More than high school	299 (46.1%)	19 (44.2%)		
**Has a form of insurance**				
No	132 (21.6%)	13 (32.5%)	1.00	0.109
Yes	479 (78.4%)	27 (67.5%)	0.57 (0.29, 1.14)	
**Used government assistance in past year**				
No	409 (64.0%)	26 (61.9%)	1.00	0.784
Yes	230 (36.0%)	16 (38.1%)	1.09 (0.57, 2.08)	
**Binge drinking in past 2 months**				
No	436 (67.4%)	36 (81.8%)	1.00	**0.047**
Yes	211 (32.6%)	8 (18.2%)	0.46 (0.21, 1.01)	
**Drug use in past 2 months**				
No	201 (31.4%)	14 (31.8%)	1.00	0.949
Yes	440 (69.6%)	30 (68.2%)	0.98 (0.51, 1.89)	
**Marijuana Use Only in past 2 months**				
No drug use	201 (35.3%)	14 (35.9%)	1.00	0.936
Yes	369 (64.7%)	25 (64.1%)	0.97 (0.49, 1.91)	
**Age at sexual debut** (median, range)	15.0 (11.0)	15.0 (10.0)	1.04 (0.90, 1.20)	0.994
**Consistent Condom Use in past 2 months**				
No	374 (62.0%)	27 (65.9%)	1.00	0.624
Yes	229 (39.0%)	14 (34.1%)	0.85 (0.43, 1.65)	
**Number of Female Sexual Partners in past 2 months**				
One female sexual partner	391 (60.2%)	26 (59.1%)	1.05 (0.56, 1.95)	0.880
Two or more partners	258 (39.8%)	18 (40.9%)		
**Total Lifetime Sexual Partners** (median, range)	6.0 (90.0)	6.0 (70.0)	0.99 (0.96, 1.02)	0.601
**Male Partners in Lifetime**				
No	607 (96.5%)	38 (88.4%)	1.00	**0.009**
Yes	22 (3.5%)	5 (11.6%)	3.63 (1.30, 10.12)	
**Prior Incarceration**				
No	472 (75.8%)	26 (61.9%)	1.00	**0.045**
Yes	151 (24.2%)	16 (38.1%)	1.92 (1.00, 3.68)	
**Prior Ct test**				
No	238 (36.7%)	13 (29.5%)	1.00	0.341
Yes	411 (63%)	31 (70.5%)	1.38 (0.71, 2.69)	
**Prior GC test**				
No	295 (57.8%)	24 (72.7%)	1.00	0.092
Yes	215 (42.2%)	9 (27.3%)	0.51 (0.23, 1.13)	
**Prior syphilis test**				
No	357 (70.0%)	26 (78.8%)	1.00	0.283
Yes	153 (30.0%)	7 (21.2%)	0.63 (0.27, 1.48)	
**Ct/GC positive during study**				
No	567 (87.4%)	40 (90.9%)	1.00	0.490
Yes	82 (12.6%)	4 (9.1%)	0.69 (0.24, 1.98)	

## Discussion

We found that, while the majority of young Black MSW had ever been HIV tested, few of those who were eligible were tested in the last year. HIV testing was associated in bivariate analysis with factors that might increase the risk of acquisition of these infections, such as substance use and inconsistent condom use, which indicates that testing efforts to reach high risk persons are reaching these individuals. However, there were still significant gaps in testing, compared to testing guidelines. Fewer than half of those meeting the guidelines for annual HIV screening, had received a test in the previous year. This may indicate that a significant proportion of individuals are not being screened as recommended, which may be contributing to the disproportionate burden of transmission among young Black people residing in the Deep South.

Similar associations were seen both for testing in the lifetime and testing in the last year if recommended, where age and prior Ct, GC, or syphilis testing were significant in multivariable modeling. However, incarceration was not significantly associated with being tested in the last year in multivariable modeling, though incarceration was significantly associated with ever being tested. Interestingly, older age at sexual debut was associated with greater odds of being HIV tested in the prior year if recommended for annual screening, despite younger age at sexual debut normally being a proxy for HIV greater risk [16]. This might be due to individuals who delayed sexual debut being more likely to be compliant with testing recommendations. However, age of sexual debut was not significant in multivariable modeling, meaning that it may also be confounded by another factor, such as STI test receipt.

The results seen in this study are similar to those that have been published elsewhere [17]. One study examining HIV risk among heterosexual males and females at elevated HIV risk across 23 geographic areas of the US, found that among all males, 75.6% had been tested in their lifetimes and 38.0% had been tested in the previous year [17]. Moreover, in that study, of all participants aged 18–24 (including both males and females), 60.2% had been tested in their lifetimes and 40.6% had been tested in the previous year [17]. Comparatively, our study found that 52.2% of participants reported ever having been HIV tested, and 41.7% reported having been tested in the past year. Hence, the testing rates are approximately what would be presumed based on national estimates, though lifetime testing is slightly lower. In contrast, the rate of reported positivity in our study was 6.3%, which far exceeds the 2020 positivity estimate of 0.5% [18]. Hence, these findings likely indicate that the convenience sample presently used is at elevated HIV risk compared to the general population, as thus may have self-selected into this study.

Our study findings can also be contextualized by other work examining barriers to testing among Black men, particularly in the Deep South. One qualitative study, which included heterosexual Black men in North Carolina found that barriers to testing included concerns about confidentiality and issues of mistreatment by healthcare providers [19]. Studies have also found a preference for community-based or decentralized HIV testing, and that only being able to test in a facility may pose a barrier [19, 20]. In contexts outside of the Deep South, stigma and discrimination, as well as lack of access to healthcare have also been described as barriers to HIV testing [21–23]. 

Our study findings add to the need for interventions to address previously identified barriers to testing. Having a prior Ct, GC, or syphilis test was associated with HIV testing, indicating that providers are likely ordering these tests together, or for the same individual, meaning that if individuals are engaged in care, they may be receiving comprehensive screening. Additionally, most of the men enrolled in the study had some form of insurance; however, other barriers to testing may remain. Hence, understanding factors leading to disclosure of HIV and sexual health risks and provider access in this population may be beneficial in increasing HIV testing.

There are several strengths and limitations to consider in the interpretation of our results. It is likely that those with a prior HIV diagnosis may be more interested in STI testing, and thus disproportionately self-selected into the study. Given that this study used convenience sampling, rather than probability sampling, inferences cannot be made about the community prevalence of HIV infection. Additionally, we were unable to determine who shared injection drug use, and thus would be recommended for annual screening for this reason; however, studies have demonstrated high levels of sharing of injection drug use supplies [24], and given that few individuals injected drugs in this study, this was unlikely to bias results. Moreover, it is important to note that only self-reported data was used in this study. It is possible that the individuals in this study over-reported their test positivity or that there was recall bias, where those with a positive test were more likely to remember being tested and report their results. However, HIV positivity was associated with having male partners; thus, it is likely that these results are reliable, given that they are aligned with expected risk factors. Moreover, the reported HIV testing rates were similar to those seen in national studies [17]. Additionally, 73.0% percent of participants in this sample had received sexual education, which included information on STIs in 90.5% of cases [25]; hence, questions around HIV are presumed to be well understood among this population. Finally, the use of computer-assisted, self-administered surveys would minimize social-desirability bias in reporting HIV/STI history.

In conclusion, this study demonstrated that self-reported HIV screening is lower than recommended among young Black men who have sex with women in the Deep South. Given the high rates of undiagnosed HIV, efforts should be made to engage individuals, such as young Black cisgender men who have sex with women at elevated risk in screening, particularly in the Deep South, which faces a disproportionate burden of HIV/STI. This would support the Ending the Epidemic initiative goals in decreasing the rate of undiagnosed infection, particularly in priority areas [26].

### Electronic supplementary material

Below is the link to the electronic supplementary material.

Supplementary Material 1
