# Supplementary material for: HIV Screening Among Young Black Men Who Have Sex with Women in New Orleans, LA
Source: AIDS Behav. 2024 May 7;28(8):2821–8. doi: 10.1007/s10461-024-04354-7 (PMC11286674; doi:10.1007/s10461-024-04354-7)
Supplement: Supplementary file 1 — Supplementary Material 1 [file 10461_2024_4354_MOESM1_ESM.docx]

**Table S1: Multivariable Regression Model with Outcome of Lifetime HIV Testing**

| Factor | Adjusted OR (95% CI) | p-value |
| --- | --- | --- |
| Age (per year older) | 1.27 (1.19, 1.34) | < 0.001 |
| Prior Ct, GC, and/or syphilis test | 6.45 (4.85, 8.57) | < 0.001 |
| Prior incarceration | 1.70 (1.16, 2.48) | 0.006 |

**Table S2: Multivariable Regression Model with Outcome of Annual HIV Testing among those Recommended**

| Factor | Adjusted OR (95% CI) | p-value |
| --- | --- | --- |
| Age (per year older) | 1.19 (1.10, 1.29) | < 0.001 |
| Prior Ct, GC, and/or syphilis test | 6.00 (4.10, 8.78) | < 0.001 |

**Table S3: Multivariable Regression Model with Outcome of HIV Positivity**

| Factor | Adjusted OR (95% CI) | p-value |
| --- | --- | --- |
| Male partner in lifetime | 3.63 (1.30, 10.12) | 0.014 |
